# Association between short-term exposure to air pollution and COVID-19 mortality in all German districts: the importance of confounders

**DOI:** 10.1186/s12302-022-00657-5

**Published:** 2022-08-27

**Authors:** Gregor Miller, Annette Menzel, Donna P. Ankerst

**Affiliations:** 1 Department of Mathematics, Technical University of Munich, Boltzmannstrasse 3, Garching, Germany; 2 Department of Life Science Systems, Technical University of Munich, Freising, Germany

**Keywords:** Variable selection, COVID-19, Air quality, Pollution, Change-in-estimate, LASSO, AIC, BIC, Cross-sectional

## Abstract

**Background:**

The focus of many studies is to estimate the effect of risk factors on outcomes, yet results may be dependent on the choice of other risk factors or potential confounders to include in a statistical model. For complex and unexplored systems, such as the COVID-19 spreading process, where a priori knowledge of potential confounders is lacking, data-driven empirical variable selection methods may be primarily utilized. Published studies often lack a sensitivity analysis as to how results depend on the choice of confounders in the model. This study showed variability in associations of short-term air pollution with COVID-19 mortality in Germany under multiple approaches accounting for confounders in statistical models.

**Methods:**

Associations between air pollution variables PM_2.5_, PM_10_, CO, NO, NO_2_, and O_3_ and cumulative COVID-19 deaths in 400 German districts were assessed via negative binomial models for two time periods, March 2020–February 2021 and March 2021–February 2022. Prevalent methods for adjustment of confounders were identified after a literature search, including change-in-estimate and information criteria approaches. The methods were compared to assess the impact on the association estimates of air pollution and COVID-19 mortality considering 37 potential confounders.

**Results:**

Univariate analyses showed significant negative associations with COVID-19 mortality for CO, NO, and NO_2_, and positive associations, at least for the first time period, for O_3_ and PM_2.5_. However, these associations became non-significant when other risk factors were accounted for in the model, in particular after adjustment for mobility, political orientation, and age. Model estimates from most selection methods were similar to models including all risk factors.

**Conclusion:**

Results highlight the importance of adequately accounting for high-impact confounders when analyzing associations of air pollution with COVID-19 and show that it can be of help to compare multiple selection approaches. This study showed how model selection processes can be performed using different methods in the context of high-dimensional and correlated covariates, when important confounders are not known a priori. Apparent associations between air pollution and COVID-19 mortality failed to reach significance when leading selection methods were used.

**Supplementary Information:**

The online version contains supplementary material available at 10.1186/s12302-022-00657-5.

## Background

In light of the worldwide impact of COVID-19 ubiquitously on society sectors, an increasing supply of studies have been conducted to ascertain the risk factors shaping the spread and severity of the disease. Studies based on aggregated and individual-level data have identified a multitude of clinical and demographic risk factors, including age [1–4], gender [2–5], ethnicity [5], income [5, 6], education [5], mobility [7], obesity [4, 8–10], hypertension [3, 4, 10–12], cardiovascular disease [2, 11, 12], respiratory disease [4, 12], pneumonia [1, 2, 4], history of cancer [10, 13], diabetes [3, 4, 10–12], and chronic kidney disease [4, 14, 15]. The high number of potential confounders in COVID-19 studies and large heterogeneity in approaches to adjust for them warrants robustness strategies. Air quality is one of the factors that are hypothesized to play a role, however, the overall results of previous studies are heterogeneous.

To illustrate the issues, this study focuses on the relationship between air quality and COVID-19 as one of the pressing environmental concerns with COVID-19-related morbidity and mortality. Pollution is the largest environmental cause of death being responsible for 16% of all deaths worldwide [16]. Exposure to air pollution increases the risk for hospital admission for cardiovascular and respiratory diseases [17] and enhances general mortality [18]. The predominant effect of COVID-19 on the lower and upper respiratory tract [19] can be anticipated to be compounded by the additional targeted effects of air pollution and individual risk factors, such as smoking and cardiovascular disease history, leading to multiple pathways impacting patient outcomes. For example, pollution could affect susceptibility to COVID-19 via the increase of hypertension and cardiovascular diseases [20] or weaken the host defense system of the respiratory system [21, 22]. Airborne particles might serve as carriers for pathogens, thereby supporting the dominant airborne transmission [23, 24]**.** Multiple studies have analyzed aspects of the association between air quality and COVID-19 outcomes, including infections and mortality (Table 1). Some studies did not account for any confounders, others only for a small fixed set. Studies adjusting for wider ranges of confounders more often failed to find significant associations.

**Table 1:** Overview of selected publications studying associations between air quality and COVID-19 statistics

Study	Approach	Result	Area	Time
Ogen [61]	Categorized NO_2_ measurements were compared	The results indicated a strong association between high values of the pollutant and high fatality cases	66 administrative regions in Italy, Spain, France, and Germany	January to February 2020
Bashir et al. [62]	The individual correlation between risk factors and new infections, total infections, and mortality were measured on a daily basis. Kendall and Spearman rank correlation was calculated. It is not clear what measurement was used to determine air quality	Besides temperature, air quality was significantly correlated with the COVID-19 metrics	New York City, USA	March to April 2020
Accarino et al. [63]	The Spearman correlation between PM_2.5_*,* PM_10_*,* NO_2_ and COVID-19 incidence rate as well as mortality rate was measured	Significant associations between all of them were found	107 Italian territorial areas	February and March 2020
Zhu et al. [64]	Daily infections, meteorological variables, and air pollution concentrations for PM_2.5_, PM_10_, SO_2_, CO, NO_2_, and O_3_ were collected. Generalized additive models were used to estimate the associations between lagged, moving average concentrations of air pollutants and daily infections	Significant positive associations for PM_2.5_, PM_10_, CO, NO_2_, and O_3_ and a negative association for SO_2_ were shown	120 Chinese cities	January to February 2020
Stieb et al. [41]	A negative binomial model was used to measure the association between PM_2.5_ from 2000 to 2016 and infection count. The Akaike information criterion was used to some extent to select from the socio-demographic, health, time since peak incidence, and temperature variables	The multivariate model did not show a significant association for PM_2.5_	111 Canadian regions	Up to May 13, 2020
Wu et al. [65]	Negative binomial mixed models were used to regress on the mortality rate with PM_2.5_ and 20 other confounders as predictors. The particulate matter between 2000 and 2016 was considered	A notable association was found for PM_2.5_, population density, days since first reported case, household income, percent of owner-occupied housing, high school education, age, and percent of Black residents	3089 US counties	Up to June 18, 2020
Rodriguez-Villamizar et al. [42]	A negative binomial hurdle model was used to analyze the effect of PM_2.5_ measured between 2014 and 2018 on COVID-19 mortality including socio-demographic, socio-economic and health confounders	PM_2.5_ did not show a significant association with mortality	772 Colombian municipalities	Up to July 17, 2020
Adhikari et al. [43]	A negative binomial regression was applied on time-series data. Besides daily PM_2.5_ and ozone, meteorological confounders were included	Ozone was found to be significantly associated with the daily infections but not with deaths	Queens county, New York, USA	March to April 2020
Borro et al. [66]	Simple linear regressions were performed for cumulative COVID-19 incidence, mortality rate, and case-fatality rate with PM_2.5_ as predictor	Significant associations were found for all three metrics	110 Italian provinces	February to March 2020
Travaglio et al. [44]	Negative binomial models were used to measure the association between PM_2.5_, PM_10_, NO, NO_2_, O_3_ and COVID-19 cases as well as deaths. Population density, average age, and mean earning were included as confounders. Air quality data prior to the pandemic were aggregated over one and five years	Both COVID-19 metrics showed significant associations with the air quality risk factors	England on regional and sub-regional level	February to May 2020
Tieskens et al. [67]	The incidence of five distinct time periods was analyzed via mixed-effect Poisson regression. Besides PM_2.5_, also 19 other socio-demographic, occupational, and mobility variables were incorporated. The variables were selected by excluding covariates with a variance inflation factor higher 2.5 in the regression of the first time period	PM_2.5_ was not selected, yet almost all selected socio-demographic and economic variables indicated strong variance of their association between the time periods	351 cities in Massachusetts, USA	March to October 2020
Liang et al. [45]	Zero-inflated negative binomial models were used to determine the association between NO_2_, PM_2.5_, and O_3_ and case-fatality and mortality rates. Air quality measurements between 2010 and 2016 were considered. The models also included socio-demographic, socio-economic, health, and mobility variables	For NO_2_, a positive association with the COVID-19 metrics was found	3122 US counties	January to July 2020

Targeted association analyses, such as between air pollution and COVID-19 outcomes here, aim to both accurately estimate independent effect sizes as well as determine statistical significance. Determination of independent effects of a risk factor of primary interest requires adjustment for all potential confounders, many of which may be related. Although the liberal use of data-driven variable selection methods to control for confounders has been criticized [25–27], such methods remain in widespread use. Among four major epidemiological journals in 2015, half used prior or causal graphs to select variables, 12% a change in effect estimate approach, 9% stepwise methods, 5% univariate analyses, 2% other methods, and 37% did not report their methods in detail [28]. Within any given study, robustness of primary association analyses to choice of confounders for inclusion is typically omitted, although sensitivity to choice of confounders has been demonstrated even in cases of small numbers of confounders [29]. The objective of this study was to compare the resulting associations of air pollution with COVID-19 mortality in high-dimensional settings when applying leading epidemiological methods for confounder selection.

## Methods

The outcomes of interest were cumulative COVID-19 mortality counts from the 400 districts in Germany for two time periods, March 2020–February 2021 and March 2021–February 2022, extracted from the Robert Koch-Institut [30] (dl-de/by-2-0 [31]) (Additional file 1: Figure S1). During the analyzed timeframe, the local health departments of two districts, Eisenach and Wartburgkreis, merged and thus merged their COVID-19 numbers. The two time periods were selected to reflect an initial phase, where lockdowns led to decreased mobility and pollution, and a later re-opening phase with increased levels. The advantage of a single country analysis is that the availability and measuring processes for the observed data are standardized at least to a certain degree, which is especially relevant with respect to the international and temporal differences in reporting COVID-19 statistics [32, 33]. Furthermore, Germany, which holds the largest population in Europe, provides extensive data on potential confounders with high spatial resolution. COVID-19 death counts were used as the outcome as there is considerable, fluctuating under-ascertainment for infection counts. Even though also undercounted and varying with time, mortality data are considered more complete than infection data [32, 33].

### Risk factors

For association with cumulative COVID-19 counts, 43 risk factors were assembled for the 400 German districts over the two time periods (Additional file 1: Table S1). Daily CO, NO, NO_2_, O_3_, PM_10_, and PM_2.5_ measurements extracted from the ENSEMBLE dataset of the Copernicus Atmosphere Monitoring Service referred to surface estimates at noon with a 0.1° horizontal coverage over all of Germany [34]. The ENSEMBLE dataset extracts the value from nine numerical air quality models and thereby achieves a higher degree of robustness than individual models. Daily district-wide estimates of the air quality values from extracted polygons were aggregated by calculating the weighted mean depending on how much of the respective district area was covered by the corresponding polygon. For each of the two analyzed time periods and each district, the average of the daily values was then calculated for inclusion as risk factors in the models.^1^

Socio-demographic, health infrastructure, political, educational, and socio-economic variables were extracted from the German Federal and State Statistical Offices [35, 36] (license: dl-de/by-2-0 [31], tables: 12411-0015, 11111-0002, 12411-0018, 12521-0040, 12521-0041, 12531-0040, AI014-1, AI014-2, AI003-2, AI005, AI-N-01-2, AI-N-10, AI-S-01, AI007-1). Political variables referred to the federal election in 2017; gross domestic product, disposable income, and employee distribution referred to 2018; education level, socio-demographic, proportion of settlement and traffic area, and health infrastructure, 2019, except for hospital bed density in 2017. Geographic data on district area were acquired from the OpenDataLab [37] (Geodatenzentrum © GeoBasis-DE/BKG 2018 (VG250 31.12., Data changed)).

Daily mobility data were extracted from the Google Community Mobility Report [38] and was only available on a state level for the 16 states in Germany. Mobility data quantified change in number of visits and length of stay for certain places, including groceries, pharmacies, parks, residences, retail and recreational areas, transit stations, and workplaces, with respect to a reference period between January 3 and February 6, 2020. Daily values were averaged over respective time periods. Flu and vaccination data were extracted from the Robert Koch-Institut [39, 40] (dl-de/by-2-0 [31]). Means of the reported yearly flu incidences between 2017 and 2019 were calculated for each district. Daily vaccination rates reported the number of people who had received full vaccination status in the district of vaccination divided by the population of the corresponding district. Vaccination rates at the end of the respective period were used for analysis. Finally, the mean of the reported yearly flu incidences between 2017 and 2019 was calculated for each district.

### Statistical methods

Two-sample *t*-tests were used to assess differences in risk factors between the two time periods, with two-sided 0.05 levels considered statistically significant. Correlation between risk factors was assessed by the Spearman method and the corresponding *p*-values were approximated by using the *t*-distribution. Negative binomial regression was used for the univariate and multivariate association analyses of risk factors with cumulative COVID-19 mortality counts, with the logarithm of the population size as offset. Negative binomial regression extends the variation of Poisson regression to accommodate overdispersion, and hence is commonly used in COVID-19 studies [41–45]. The exponentiated coefficient estimates of the negative binomial model are called incidence rate ratios (IRR).

Due to the high correlation between some of the air pollution variables, CO, NO, NO_2_, O_3_, PM_10_, and PM_2.5_, each was analyzed separately. A literature search identified leading methods for variable selection, which were investigated in the study as part of a sensitivity analysis [28, 46, 47]. Additionally, basic and full models were analyzed, either including only the considered air pollution variables, or all other risk factors as well. Selection methods were applied such that the respective air pollution variable, the target parameter, was always included in the model. This separates the approaches of this paper from other applications concerned only with prediction or interest in all risk factor effects equally.

Variable selection and model fitting, including basic and full models, were performed utilizing bootstrap sampling with 100 samples to obtain confidence intervals of coefficient estimates and included covariate numbers using quantiles [48]. All calculations were implemented using R version 4.1.2 [49] including the packages MASS [50], furrr [51], mpath [52], Hmisc [53], and lmtest [54].

### Selection methods

The traditional stepwise selection method based on significance uses *p*-values to determine if the corresponding covariate should be included in the model. In this study, the selection criterion *p* < 0.05 was used. For the forward approach, the starting model is the basic model only including the current air pollution covariate. Iteratively a single new variable at a time is included in the current model. Each of the new models is compared to the current model via the likelihood ratio test, selecting the model with the smallest *p*-value. The process is repeated until all of the new potential models have *p* ≥ 0.05 or all of the potential covariates are included. In the backwards variant, the full model is the starting model and variables are discarded when their exclusion leads to the largest *p*-value. The process is stopped if all new potential models have *p* < 0.05 or only the air pollution covariate remains. The problem of the significance approach is that it can only determine if a risk factor is relevant given the other risk factors incorporated in the model.

Again starting with the basic or full model according to the forwards or backwards specification, also the Akaike (AIC) and Bayesian Information Criterion (BIC) were used. In this case, the models were selected with the smallest AIC or BIC value, respectively. With these criteria, it was possible to consider not only either inclusion or exclusion of covariates when comparing models, but both, regardless of the initial model. In general, the BIC penalizes larger numbers of covariates more severely and therefore favors smaller models. Information criteria allow the user to sort through huge numbers of models, while being computationally very efficient. However, as any of the stepwise approaches, they do not guarantee stable results such that small changes in the data may lead to very different selections.

In the change-in-estimate method (CIE), the selection criterion is based on the change of the coefficient estimate of the target parameter, in our case the air pollution variable. The implementation of the method occurs in many different flavors. In the predominant variant [55], a full model is fitted including the target parameter and all possible confounders. Confounders are then removed from the model one at a time until it becomes impossible to remove a confounder without altering the target parameter effect estimate too much compared to the estimate produced by the initial model. The change-in-estimate is defined as:

$$\Delta \mathrm{CE}=\frac{\left| {\mathrm{CE}}_{i}- {\mathrm{CE}}_{0} \right|}{{\mathrm{CE}}_{0}},$$

where ${\mathrm{CE}}_{i}$ is the target parameter effect estimate of the considered model with one of the confounders removed and ${\mathrm{CE}}_{0}$ is the estimate of the initial model. In this backward variant, the confounder leading to the smallest change is selected as long as it is smaller than ten percent. A different option is the forward approach, where the initial model is the basic model including no confounders and confounders leading to the largest change are added as long as the change-in-estimate is larger than ten percent. The variant, where the change-in-estimate is not calculated with respect to the estimate of the initial model but with respect to the estimate of the model of the previous step, was also considered. The CIE approach offers an intuitive way to exclude and include risk factors in a model based directly on the changes in the coefficient estimates; however, setting the threshold of decision may even be more arbitrary than in other methods.

Finally, a variable selection, which is usually not presented as part of the traditional selection methods but has found its use in various studies, was also implemented [56, 57]. In this approach, the least absolute shrinkage and selection operator (LASSO) is used to select the relevant variables. LASSO is a shrinkage estimator penalizing the likelihood, thereby shrinking some coefficient estimates to zero. As all coefficient estimates are biased, the non-zero coefficients are then selected and used to refit the model to receive interpretable coefficient estimates. Cross-validation was used to set the hyperparameter of the procedure and no penalty factor for the air pollution variable was set to guarantee that it stayed in the model. The shrinkage approach of LASSO allows a more robust selection of risk factors than the other methods; however, it prohibits the direct interpretation of coefficient estimates.

## Results

Before comparing the model selection approaches to evaluate the impact of the air pollution effects on COVID-19 mortality, the data are first visually and quantitatively explored. Comparisons between the first and second year after the start of the COVID-19 pandemic (Additional file 1: Table S1) showed that the air pollution variables all increased significantly except for ozone, which showed a significant decrease (all *p* < 0.001). NO, NO_2_, PM_2.5_, and PM_10_ more than doubled in the second period. Visits to grocery stores and pharmacies increased in comparison to the reference in the first year (median of district values: 16.5%), this dropped down again in the second year (7.0%, *p* < 0.001). Activity in parks remained on an increased level (57.0% and 58.5%), while activity in transit stations and at workplaces decreased in the first period (− 13.5% and − 2.0%) and then dropped even further (− 31.0% and − 26.0%, both *p* < 0.001). While the number of infections increased from the first to second period (27.9 to 151.1 infections per 1000 inhabitants, *p* < 0.001), the number of deaths decreased (86.6 to 51.5 deaths per 100 000 inhabitants, *p* < 0.001).

High correlations between some of the air pollution variables indicated the necessity to estimate their association to mortality separately (Additional file 1: Figure S2). NO_2_ and NO (Spearman rank correlation coefficient: 0.92) as well as PM_2.5_ and PM_10_ (0.91) were highly correlated. Other covariates also showed high correlations that could lead to multicollinearity. For example, transit station mobility was highly correlated with activity in retail and recreation (0.90) and workplaces (0.89), while residential and workplace mobility were negatively correlated (− 0.94). Other examples of significant correlations were between population density and proportion of urban area in a district (0.95), proportion of males and females at least 75 years old (0.91), as well as proportion of people working in the service and people working in manufacturing (− 0.99). All of these examples had *p*-values smaller than 0.0001.

The univariate analyses showed that O_3_ had a positive association with COVID-19 mortality for both considered time periods (IRR of first period: 1.02, *p*-value < 0.001; IRR second period: 1.01, *p*: 0.031) (Additional file 1: Table S2). Another significant positive association was shown for PM_2.5_ in the first period (IRR: 1.07, *p*: 0.009), this however lost significance in the second period (*p*: 0.4). Significant negative associations were estimated for NO, NO_2_, and CO in the first period (IRR: 0.90, 0.95, 1.00; *p*: 0.013, 0.002, 0.022). This remained stable for the second time period.

Many of the other covariates also showed significant associations with mortality (Additional file 1: Table S2). Generally, indicators positively associated with increased mortality included a higher proportion of older people, less foreigners, less education, more mobility in workplaces, transit stations, retail and recreation instead of residences, more votes for political parties at the outer spectrum, higher proportion of people working in manufacturing and construction, and less health personnel per persons needing inpatient care. Many of the associations remained similar between the two time periods, however, some showed changes such as the vaccination rate which was first positive (IRR: 1.23, *p*: 0.8), when barely any full vaccinations were performed, then negative one year later, although still not quite significant (IRR: 0.83, *p*: 0.07).

### Comparison of the multivariate model selection algorithms

**Fig. 1:**
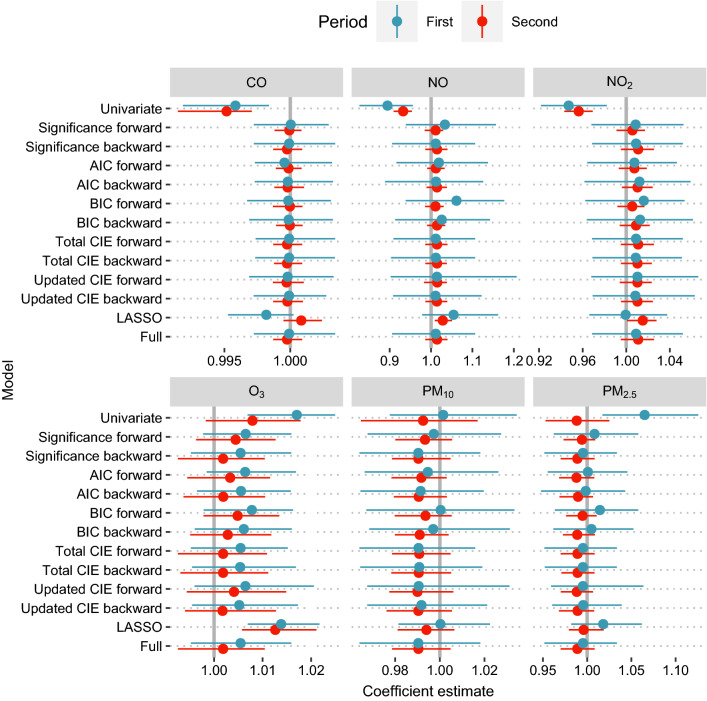
Coefficient estimates of bootstrapped variable selection processes for air pollution covariates with 95% quantiles from bootstrap samples. Generally, higher mortality rates and larger dispersion in the first period lead to increased quantiles in comparison to the second period

Coefficient estimates for all selection methods can be found in Fig. 1. The often significant association of air pollution with mortality was diminished if further variables were included. This was generally independent of the selection method. For example, NO_2_ is one of the clearest cases, where significant estimates in the univariates case were not visible anymore in the multivariate case. The coefficient estimates from first to second time period were somewhat decreased for O_3_ and PM_2.5_, otherwise, the estimates were very close between the time periods for the pollution variables, even though the variable selection methods ran independently and there were various changes for the effects of the other covariates. Another important result was that, for most selection methods, the coefficient estimates were equivalent to the full model. The LASSO selection as the only non-standard method led to larger deviations and sometimes did not converge properly. The otherwise homogeneity between the selection methods, however, did not translate to the number of selected covariates. In addition, multivariate analyses were performed for all air quality metrics and selection methods except LASSO with two additional risk factors, temperature and precipitation, which yielded similar results and were therefore not considered further.

**Fig. 2:**
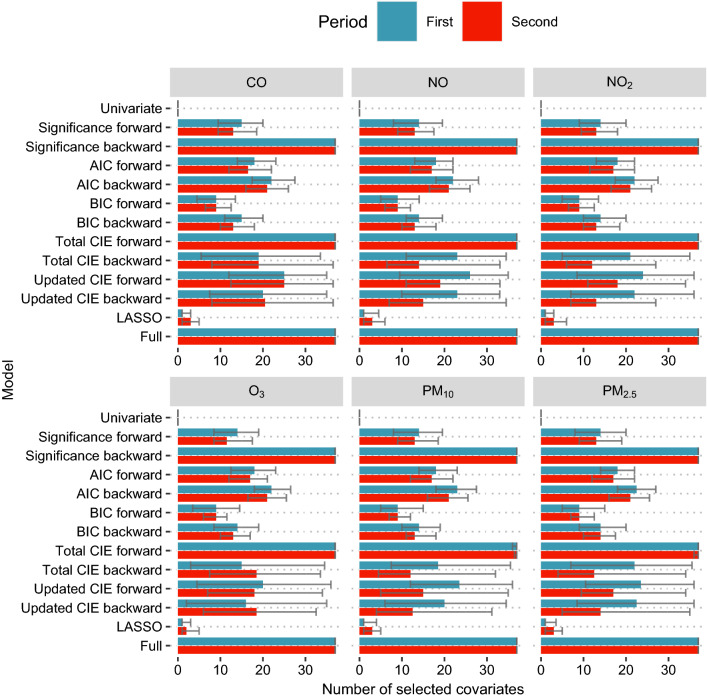
Median number of selected confounders after variable selection process with 95% quantiles from bootstrap samples

**Fig. 3:**
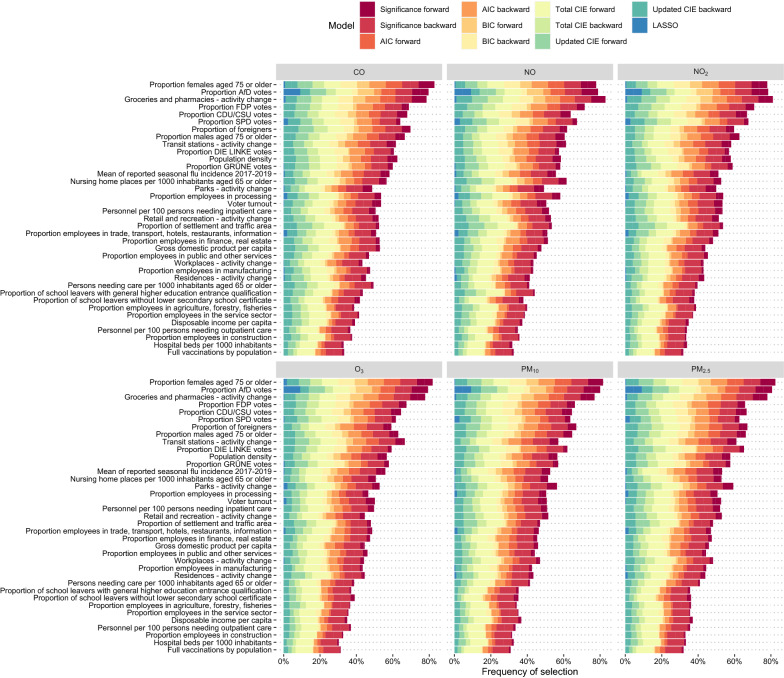
Selection frequency of confounders depending on variable selection method aggregated for both analyzed time periods excluding the univariate and full model. For example, for CO, the proportion of females aged 75 or older was selected in 83% of the models with 8.7% being from the significance forward selection models

The number of selected covariates can be seen in Fig. 2. The BIC forward and LASSO selection methods led to the smallest number of covariates, but also showed larger differences to the full model. Almost all CIE methods had very large variances in the number of covariates, with the total CIE forward and significance backward consistently picking all covariates. Generally, the number of selected covariates was very consistent between the pollution variables. The most consistently selected covariates were the population proportion of females at least 75 years of age, the proportion of votes for the right-wing party AfD, and the activity in groceries and pharmacies, independent of the considered air pollution variable (Fig. 3).

As an example, the confidence intervals of the NO_2_ coefficient extracted as quantiles from the bootstrap estimates were also compared with those calculated analytically in a single selection run for the entire data set (Additional file 1: Table S3). The confidence intervals were extremely similar. The number of selected covariates in the single run was also very close to the median of the bootstrap results.

## Conclusion

While previous studies have investigated the impact of air pollution on COVID-19 mortality on a very short time frame with often limited confounders, leading to different conclusions, this is the first study to consider the association over two years while incorporating high dimensional confounders, as well as propose a sensitivity analysis comparing the effect of commonly proposed variable selection methods. Univariate analyses of one air pollution risk factor at a time yielded many significant results, with some pollution variables even showing negative associations with COVID-19 mortality, which failed to reach significance after adjustment for confounders by nearly all methods. One reason could be that other risk factors, such as mobility, also drive air pollution, leading to surrogacy effects. The traditional variable selection methods provided similar results and bootstrap confidence intervals were close to those of a single iteration. If there are considerable correlations of the main exposure to other risk factors, the multicollinearity effect needs to be considered and quantified. If possible, separate analyses should be considered such as in our case where separate models were created for each of the pollution variables. The analyses here demonstrate the importance of performing sensitivity analyses of targeted risk factor outcome results to multiple methods for confounder adjustment.

There are a number of limitations with respect to previous cross-sectional studies on air pollution and COVID-19, such as ignored time differences in the introduction of the virus, confounding due to aggregation of the data on a crude level [58], and omitted confounders. These vulnerabilities were avoided or at least mitigated in this study by using the highest available spatial resolution of the data and by selection of likely confounders. In this study, a single country was analyzed over a long time span starting after introduction of the virus, while many early studies considered only the first two or three months. Use of aggregated data rather than individual-level data lead to loss of specificity in risk factor outcome association precision. However, area-specific analyses are crucial to highlight the necessity of policy decisions and more feasible in the presence of large numbers of confounders, all of which could not be easily obtained for large numbers of individuals. Another limitation is that the considered air pollution metrics may be too low to measure a significant effect on the severity of COVID-19 in comparison, for example to the highly industrialized regions, Lombardy, Veneto, and Emilia-Romagna, where the initial surge of infections and deaths in Italy appeared most severely [59].

Further studies are required to determine and gauge associations of risk factors with the spread of COVID-19. Moreover, necessary data need to be available and be standardized between countries. For example, it would be necessary to know the place of residence of vaccinated people not only the place of their vaccinations, a standardized and reliable database of interventions with a high spatial resolution is necessary, and higher reliability of COVID-19 numbers is crucial. This study has focused on mortality, but when available, excess mortality with appropriate resolution should be considered as a potentially more reliable mortality measure to compare with reported deaths [60]. Comparable sensitivity analyses as performed here should be performed in other COVID-19 association studies to assess the robustness of targeted risk factor effects on outcomes, thus avoiding unnecessary or false public health actions based on spurious results.

## Supplementary Information

**Additional file 1: Figure S1.** Cumulative mortality rate and average NO_2_ in µg m^-3^ for the full considered time frame between March 2020 and February 2022 of the 400 German districts. **Figure S2.** Correlation plot of risk factors between German districts aggregated over the time frame between March 2020 and February 2022. Black borders indicate p<0.0005. **Table S1.** Risk factors and outcomes for first time period March 2020 – February 2021 and second time period March 2021 – February 2022. **Table S2.** Univariate association of variables with COVID-19 mortality for first time period March 2020 – February 2021 and second period March 2021 – February 2022. **Table S3.** Comparison of bootstrapped selection process with confidence intervals derived from the bootstrap quantiles and a single selection execution on the full dataset for NO2.

## Data Availability

All data were acquired from publicly available databases.

## Abbreviations

AIC: Akaike information criterion
BIC: Bayesian information criterion
CI: Confidence interval
CIE: Change-in-estimate
IQR: Inter-quartile range
IRR: Incidence rate ratios
LASSO: Least absolute shrinkage and selection operator

## Footnotes

1 Neither the European Commission nor the European Centre for Medium-Range Weather Forecasts are responsible for any use that may be made of the information or data this publication contains.
