# Supplementary material for: Association between short-term exposure to air pollution and COVID-19 mortality in all German districts: the importance of confounders
Source: Environ Sci Eur. 2022 Aug 27;34(1):79. doi: 10.1186/s12302-022-00657-5 (PMC9418649; doi:10.1186/s12302-022-00657-5)
Supplement: Supplementary file 1 — Additional file 1: Figure S1. Cumulative mortality rate and average NO2 in µg m-3 for the full considered time frame between March 2020 and February 2022 of the 400 German districts. Figure S2. Correlation plot of risk factors between German districts aggregated over the time frame between March 2020 and February 2022. Black borders indicate p<0.0005. Table S1. Risk factors and outcomes for first time period March 2020 – February 2021 and second time period March 2021 – February 2022. Table S2. Univariate association of variables with COVID-19 mortality for first time period March 2020 – February 2021 and second period March 2021 – February 2022. Table S3. Comparison of bootstrapped selection process with confidence intervals derived from the bootstrap quantiles and a single selection execution on the full dataset for NO2. [file 12302_2022_657_MOESM1_ESM.docx]

Additional file

Additional file: Figure 1. Cumulative mortality rate and average NO_2_ in µg m^-3^ for the full considered time frame between March 2020 and February 2022 of the 400 German districts.


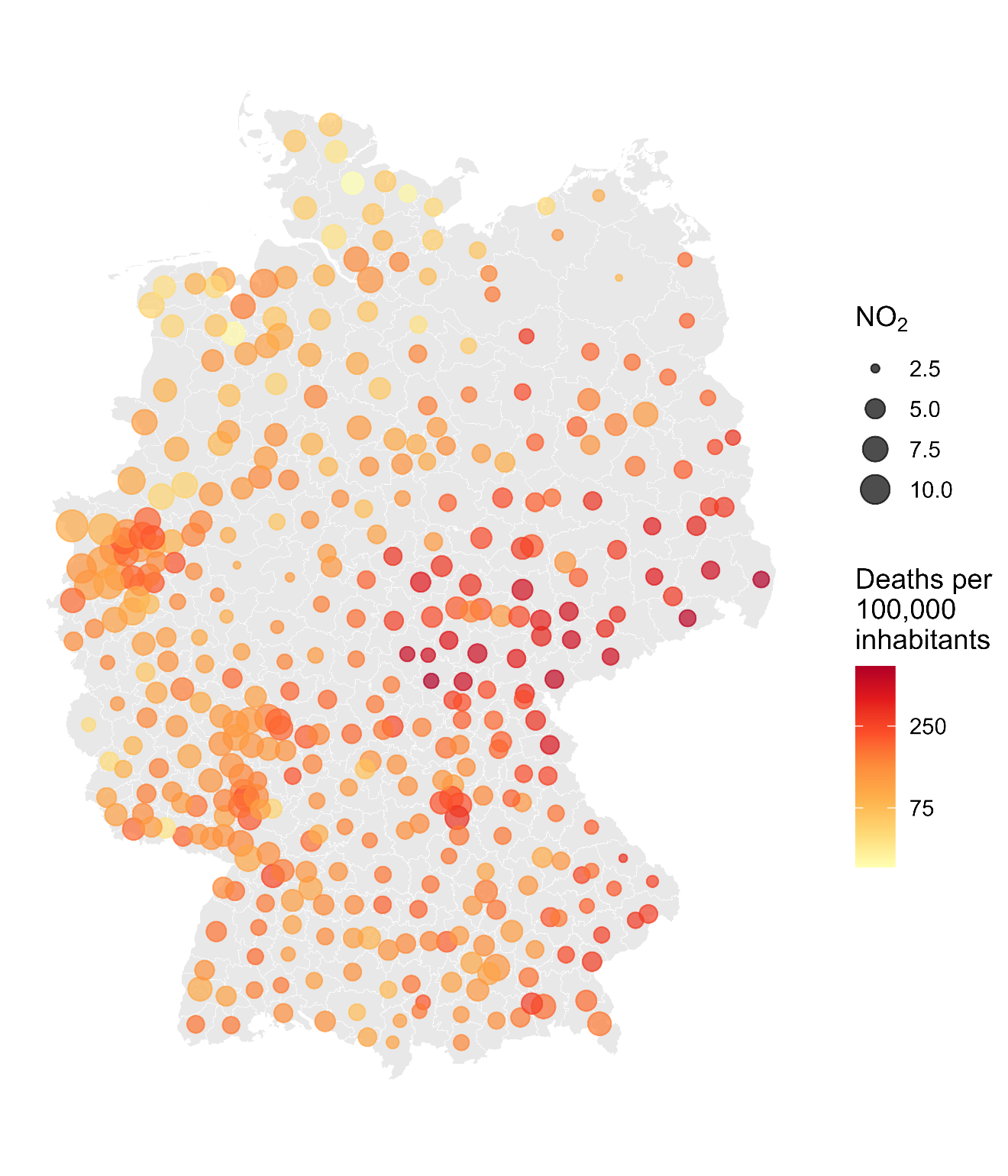


Additional file: Figure 2. Correlation plot of risk factors between German districts aggregated over the time frame between March 2020 and February 2022. Black borders indicate p<0.0005.


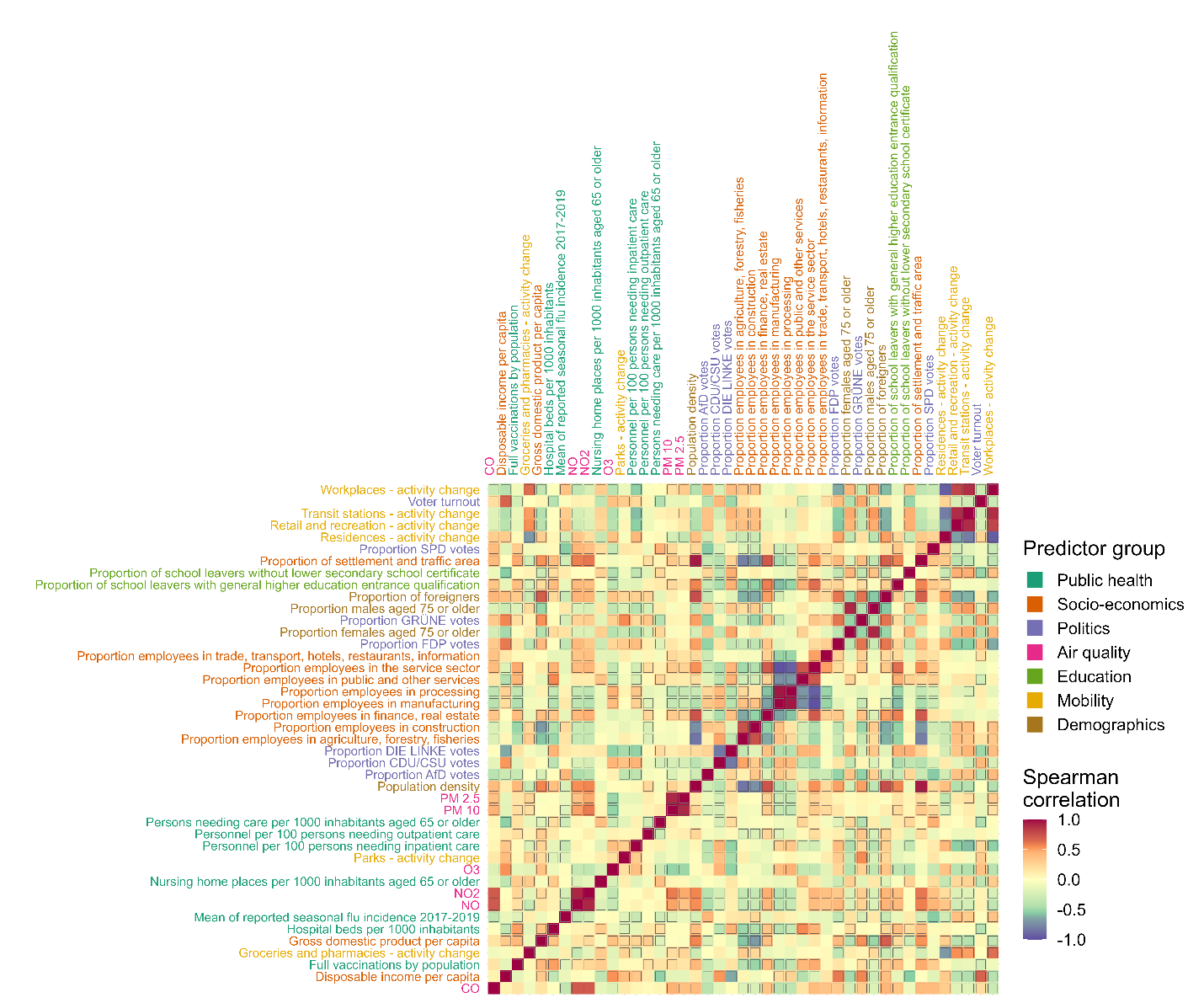


Additional file: Table 1. Risk factors and outcomes for first time period March 2020 – February 2021 and second time period March 2021 – February 2022.

|  |  | **Median (IQR)** | | **p-value** |
| --- | --- | --- | --- | --- |
|  |  | **First period** | **Second period** |  |
| **Air quality** | CO [µg m^-3^] | 166.3 (160.0, 173.3) | 203.7 (194.0, 216.0) | <0.001 |
|  | NO [µg m^-3^] | 0.9 (0.7, 1.2) | 2.3 (1.7, 3.1) | <0.001 |
|  | NO_2_ [µg m^-3^] | 3.5 (3.0, 4.6) | 7.6 (6.4, 9.5) | <0.001 |
|  | O_3_ [µg m^-3^] | 72.5 (69.4, 75.4) | 63.2 (58.4, 67.9) | <0.001 |
|  | PM_10_ [µg m^-3^] | 6.6 (5.7, 7.8) | 14.3 (12.4, 15.5) | <0.001 |
|  | PM_2.5_ [µg m^-3^] | 4.6 (4.0, 5.3) | 11.0 (9.8, 12.0) | <0.001 |
| **Demographics** | Population density | 0.2 (0.1, 0.7) |  |  |
|  | Proportion females aged 75 or older | 6.9 (6.3, 7.5) |  |  |
|  | Proportion males aged 75 or older | 4.8 (4.4, 5.2) |  |  |
|  | Proportion of foreigners | 10.6 (7.1, 15.2) |  |  |
| **Education** | Proportion of school leavers with general higher education entrance qualification | 32.5 (26.5, 38.0) |  |  |
|  | Proportion of school leavers without lower secondary school certificate | 6.5 (5.2, 8.2) |  |  |
| **Mobility** | Groceries and pharmacies - activity change | 16.5 (15.0, 20.5) | 7.0 (6.5, 10.0) | <0.001 |
|  | Parks - activity change | 57.0 (41.5, 72.5) | 58.5 (55.5, 67.5) | 0.128 |
|  | Residences - activity change | 2.0 (2.0, 2.5) | 8.0 (7.0, 8.5) | <0.001 |
|  | Retail and recreation - activity change | -15.0 (-16.0, -13.5) | -18.2 (-20.5, -16.5) | <0.001 |
|  | Transit stations - activity change | -13.5 (-13.5, -9.8) | -31.0 (-31.5, -24.0) | <0.001 |
|  | Workplaces - activity change | -2.0 (-2.0, -1.0) | -26.0 (-26.0, -17.5) | <0.001 |
| **Politics** | Voter turnout [%] | 76.1 (73.6, 78.4) |  |  |
|  | Proportion AfD votes [%] | 12.0 (9.8, 15.2) |  |  |
|  | Proportion CDU/CSU votes [%] | 33.9 (30.0, 38.7) |  |  |
|  | Proportion DIE LINKE votes [%] | 6.8 (5.7, 10.1) |  |  |
|  | Proportion FDP votes [%] | 9.9 (8.3, 11.6) |  |  |
|  | Proportion GRÜNE votes [%] | 7.3 (5.4, 10.5) |  |  |
|  | Proportion SPD votes [%] | 19.2 (15.0, 25.0) |  |  |
| **Public health** | Full vaccinations by population | 0.0 (0.0, 0.0) | 0.7 (0.7, 0.8) | <0.001 |
|  | Hospital beds per 1000 inhabitants | 5.6 (3.7, 8.2) |  |  |
|  | Mean of reported seasonal flu incidence 2017-2019 | 211.8 (139.0, 356.2) |  |  |
|  | Nursing home places per 1000 inhabitants aged 65 or older | 54.7 (48.0, 61.5) |  |  |
|  | Personnel per 100 persons needing inpatient care | 96.5 (89.7, 106.6) |  |  |
|  | Personnel per 100 persons needing outpatient care | 40.4 (35.2, 46.7) |  |  |
|  | Persons needing care per 1000 inhabitants aged 65 or older | 226.5 (198.4, 252.3) |  |  |
| **Socio-economics** | Disposable income per capita | 22564.0 (20763.0, 24283.5) |  |  |
|  | Gross domestic product per capita | 33636.0 (28131.0, 40608.0) |  |  |
|  | Proportion employees in agriculture, forestry, fisheries | 1.7 (0.5, 3.0) |  |  |
|  | Proportion employees in construction | 6.4 (4.8, 8.1) |  |  |
|  | Proportion employees in finance, real estate | 13.1 (11.0, 16.2) |  |  |
|  | Proportion employees in manufacturing | 26.7 (20.3, 34.2) |  |  |
|  | Proportion employees in processing | 18.1 (12.8, 25.4) |  |  |
|  | Proportion employees in public and other services | 31.0 (27.1, 36.2) |  |  |
|  | Proportion employees in the service sector | 70.1 (63.3, 77.7) |  |  |
|  | Proportion employees in trade, transport, hotels, restaurants, information | 24.3 (22.1, 27.3) |  |  |
|  | Proportion of settlement and traffic area | 14.3 (11.2, 29.3) |  |  |
| **Outcome** | Deaths per 100,000 inhabitants | 86.6 (62.6, 119.5) | 51.5 (36.9, 74.7) | <0.001 |

The number of deaths is the outcome. The p-values indicate the difference of the values between first and second period according to the univariate, two-sided t-test. If the values are missing for the second period, they are equal to the first period. For three variables, up to ten districts had missing values, in education, politics, public health, and socioeconomics single districts had missing values, all other variables had full data. IQR: interquartile range.

Additional file: Table 2. Univariate association of variables with COVID-19 mortality for first time period March 2020 – February 2021 and second period March 2021 – February 2022.

|  | | **First period** | | **Second period** | |
| --- | --- | --- | --- | --- | --- |
|  |  | **IRR (95% CI)** | **p-value** | **IRR (95% CI)** | **p-value** |
| **Air quality** | O_3_ | 1.02 (1.01, 1.03) | <0.001 | 1.01 (1.00, 1.02) | 0.031 |
|  | NO_2_ | 0.95 (0.92, 0.98) | 0.002 | 0.96 (0.94, 0.97) | <0.001 |
|  | PM_2.5_ | 1.07 (1.02, 1.12) | 0.009 | 0.99 (0.96, 1.02) | 0.387 |
|  | NO | 0.90 (0.83, 0.97) | 0.013 | 0.93 (0.90, 0.96) | <0.001 |
|  | CO | 1.00 (0.99, 1.00) | 0.022 | 1.00 (0.99, 1.00) | <0.001 |
|  | PM_10_ | 1.00 (0.98, 1.03) | 0.780 | 0.99 (0.97, 1.01) | 0.290 |
| **Demographics** | Proportion females aged 75 or older | 1.22 (1.17, 1.27) | <0.001 | 1.29 (1.24, 1.35) | <0.001 |
|  | Proportion males aged 75 or older | 1.30 (1.21, 1.39) | <0.001 | 1.42 (1.32, 1.52) | <0.001 |
|  | Proportion of foreigners | 0.98 (0.98, 0.99) | <0.001 | 0.98 (0.97, 0.98) | <0.001 |
| **Education** | Proportion of school leavers without lower secondary school certificate | 1.04 (1.02, 1.06) | 0.001 | 1.07 (1.04, 1.09) | <0.001 |
|  | Proportion of school leavers with general higher education entrance qualification | 0.99 (0.98, 1.00) | 0.001 | 0.98 (0.98, 0.99) | <0.001 |
| **Mobility** | Residences - activity change | 0.59 (0.54, 0.65) | <0.001 | 0.88 (0.83, 0.93) | <0.001 |
|  | Workplaces - activity change | 1.12 (1.09, 1.14) | <0.001 | 1.04 (1.03, 1.05) | <0.001 |
|  | Transit stations - activity change | 1.03 (1.02, 1.04) | <0.001 | 1.03 (1.02, 1.03) | <0.001 |
|  | Retail and recreation - activity change | 1.05 (1.03, 1.06) | <0.001 | 1.07 (1.06, 1.09) | <0.001 |
|  | Groceries and pharmacies - activity change | 1.02 (1.00, 1.03) | 0.020 | 0.98 (0.96, 1.00) | 0.064 |
|  | Parks - activity change | 1.00 (1.00, 1.00) | 0.319 | 0.98 (0.98, 0.98) | <0.001 |
| **Politics** | Proportion AfD votes | 1.06 (1.05, 1.07) | <0.001 | 1.08 (1.08, 1.09) | <0.001 |
|  | Proportion GRÜNE votes | 0.94 (0.93, 0.95) | <0.001 | 0.92 (0.91, 0.94) | <0.001 |
|  | Proportion FDP votes | 0.93 (0.91, 0.94) | <0.001 | 0.91 (0.89, 0.92) | <0.001 |
|  | Proportion SPD votes | 0.97 (0.97, 0.98) | <0.001 | 0.95 (0.95, 0.96) | <0.001 |
|  | Proportion DIE LINKE votes | 1.04 (1.02, 1.05) | <0.001 | 1.06 (1.05, 1.07) | <0.001 |
|  | Voter turnout | 0.97 (0.95, 0.98) | <0.001 | 0.94 (0.93, 0.95) | <0.001 |
|  | Proportion CDU/CSU votes | 0.98 (0.98, 0.99) | <0.001 | 0.98 (0.97, 0.99) | <0.001 |
| **Public health** | Mean of reported seasonal flu incidence 2017-2019 | 1.00 (1.00, 1.00) | <0.001 | 1.00 (1.00, 1.00) | <0.001 |
|  | Personnel per 100 persons needing inpatient care | 0.99 (0.98, 0.99) | <0.001 | 0.99 (0.98, 0.99) | <0.001 |
|  | Nursing home places per 1000 inhabitants aged 65 or older | 1.01 (1.00, 1.01) | 0.017 | 1.00 (0.99, 1.00) | 0.612 |
|  | Persons needing care per 1000 inhabitants aged 65 or older | 1.00 (1.00, 1.00) | 0.035 | 1.00 (1.00, 1.00) | 0.385 |
|  | Hospital beds per 1000 inhabitants | 1.01 (1.00, 1.03) | 0.044 | 1.02 (1.00, 1.03) | 0.020 |
|  | Personnel per 100 persons needing outpatient care | 1.00 (0.99, 1.00) | 0.178 | 1.00 (0.99, 1.00) | 0.080 |
|  | Full vaccinations by population | 1.23 (0.26, 6.78) | 0.803 | 0.83 (0.69, 1.00) | 0.067 |
| **Socio-economics** | Proportion employees in trade, transport, hotels, restaurants, information | 0.97 (0.96, 0.98) | <0.001 | 0.95 (0.94, 0.96) | <0.001 |
|  | Proportion employees in manufacturing | 1.01 (1.01, 1.02) | <0.001 | 1.02 (1.02, 1.03) | <0.001 |
|  | Proportion employees in construction | 1.05 (1.03, 1.08) | <0.001 | 1.07 (1.05, 1.10) | <0.001 |
|  | Proportion employees in the service sector | 0.99 (0.98, 0.99) | <0.001 | 0.98 (0.98, 0.99) | <0.001 |
|  | Disposable income per capita | 1.00 (1.00, 1.00) | <0.001 | 1.00 (1.00, 1.00) | <0.001 |
|  | Proportion employees in processing | 1.01 (1.00, 1.02) | <0.001 | 1.02 (1.01, 1.02) | <0.001 |
|  | Gross domestic product per capita | 1.00 (1.00, 1.00) | 0.004 | 1.00 (1.00, 1.00) | 0.009 |
|  | Proportion employees in finance, real estate | 0.99 (0.98, 1.00) | 0.038 | 0.98 (0.97, 0.99) | <0.001 |
|  | Proportion of settlement and traffic area | 1.00 (0.99, 1.00) | 0.066 | 0.99 (0.99, 1.00) | 0.001 |
|  | Proportion employees in public and other services | 0.99 (0.99, 1.00) | 0.105 | 0.99 (0.98, 1.00) | 0.003 |
|  | Proportion employees in agriculture, forestry, fisheries | 1.00 (0.96, 1.03) | 0.863 | 1.01 (0.97, 1.05) | 0.638 |

IRR: Incidence rate ratios, CI: Confidence interval.

Additional file: Table 3. Comparison of bootstrapped selection process with confidence intervals derived from the bootstrap quantiles and a single selection execution on the full dataset for NO_2_.

|  | **First period** | | | | **Second period** | | | |
| --- | --- | --- | --- | --- | --- | --- | --- | --- |
|  | **Bootstrap** | | **Single** | | **Bootstrap** | | **Single** | |
| **Model** | **Estimate (95% CI)** | **# covariates** | **Estimate (95% CI)** | **# covariates** | **Estimate (95% CI)** | **# covariates** | **Estimate (95% CI)** | **# covariates** |
| **Univariate** | 0.95 (0.92, 0.98) | 0 (0, 0) | 0.95 (0.92, 0.98) | 0 | 0.96 (0.94, 0.97) | 0 (0, 0) | 0.96 (0.94, 0.97) | 0 |
| **Significance forward** | 1.01 (0.97, 1.05) | 14 (9, 20) | 1.02 (0.99, 1.05) | 13 | 1.01 (0.99, 1.02) | 13 (9.475, 18) | 1.00 (0.99, 1.02) | 10 |
| **Significance backward** | 1.01 (0.97, 1.05) | 37 (37, 37) | 1.01 (0.97, 1.05) | 37 | 1.01 (0.99, 1.03) | 37 (37, 37) | 1.01 (1.00, 1.03) | 37 |
| **AIC forward** | 1.01 (0.96, 1.05) | 18 (13, 22) | 1.02 (0.99, 1.05) | 15 | 1.01 (0.99, 1.02) | 17 (11.475, 22) | 1.00 (0.99, 1.02) | 14 |
| **AIC backward** | 1.01 (0.96, 1.06) | 22 (17.475, 27.525) | 1.02 (0.99, 1.06) | 21 | 1.01 (1.00, 1.02) | 21 (16.475, 26) | 1.01 (1.00, 1.02) | 19 |
| **BIC forward** | 1.02 (0.96, 1.05) | 9 (5, 13.525) | 1.02 (0.99, 1.05) | 7 | 1.01 (0.99, 1.02) | 9 (6.475, 12.525) | 1.01 (0.99, 1.02) | 8 |
| **BIC backward** | 1.01 (0.96, 1.06) | 14 (10, 20) | 1.02 (0.99, 1.06) | 13 | 1.01 (0.99, 1.02) | 13 (10, 18.525) | 1.01 (1.00, 1.02) | 10 |
| **Total CIE forward** | 1.01 (0.97, 1.05) | 37 (37, 37) | 1.01 (0.97, 1.05) | 37 | 1.01 (0.99, 1.03) | 37 (37, 37) | 1.01 (1.00, 1.03) | 37 |
| **Total CIE backward** | 1.01 (0.97, 1.05) | 21 (5, 35) | 1.01 (0.97, 1.05) | 30 | 1.01 (1.00, 1.02) | 12 (6, 27) | 1.01 (1.00, 1.02) | 5 |
| **Updated CIE forward** | 1.01 (0.97, 1.07) | 24 (8.475, 36) | 1.02 (0.98, 1.07) | 21 | 1.01 (0.99, 1.02) | 18 (11, 34) | 1.01 (0.99, 1.02) | 17 |
| **Updated CIE backward** | 1.01 (0.97, 1.06) | 22 (7, 36) | 1.01 (0.97, 1.05) | 27 | 1.01 (1.00, 1.02) | 13 (7, 27) | 1.01 (0.99, 1.03) | 11 |
| **LASSO** | 1.00 (0.97, 1.04) | 1 (1, 3) | 1.00 (0.97, 1.02) | 1 | 1.02 (1.00, 1.03) | 3 (1, 6) | 1.02 (1.01, 1.03) | 3 |
| **Full** | 1.01 (0.97, 1.05) | 37 (37, 37) | 1.01 (0.97, 1.05) | 37 | 1.01 (0.99, 1.03) | 37 (37, 37) | 1.01 (1.00, 1.03) | 37 |

The number of covariates does not count the offset or the air pollution covariate.
